# Scrub typhus infection in pregnancy: the dilemma of diagnosis and treatment in a resource‐limited setting

**DOI:** 10.1002/ccr3.572

**Published:** 2016-05-06

**Authors:** Wanitda Watthanaworawit, Edyta Kolakowska, Borimas Hanboonkunupakarn, Clare Ling, Rose McGready

**Affiliations:** ^1^ Shoklo Malaria Research UnitMahidol‐Oxford Tropical Medicine Research UnitFaculty of Tropical MedicineMahidol UniversityMae SotThailand; ^2^ Mahidol‐Oxford Tropical Medicine Research UnitFaculty of Tropical MedicineMahidol UniversityBangkokThailand; ^3^ Centre for Tropical MedicineNuffield Department of MedicineUniversity of OxfordOxfordUK

**Keywords:** Acute undifferentiated fever, maternal mortality, *Orientia tsutsugamushi*, scrub typhus, sepsis, stillbirth

## Abstract

To save the life of both mother and fetus, the risks and benefits of the few antibiotics considered effective in the treatment of severe scrub typhus require consideration. In this case, chloramphenicol treatment averted maternal but not fetal mortality. Evidence‐based guidelines appropriate for resource‐limited endemic areas are required.

## Introduction

Scrub typhus caused by *Orientia tsutsugamushi*, an obligate intracellular gram‐negative bacteria, transmitted by larval trombiculid mites (chiggers), is a zoonosis endemic in the rural areas of South‐east Asia and remains a leading cause of acute undifferentiated fever (AUF) 1. Contracted during pregnancy, it may lead to adverse maternal and newborn outcomes, including an unknown burden of maternal and newborn mortality 2.

Clinical diagnosis is poor, due to the similarity in presentation with other infections commonly seen in the tropics such as typhoid, leptospirosis, and dengue fever 3, 4. In addition, a lack of sensitive and specific diagnostic tools available to rural clinics, where the disease is mostly seen, often results in under or misdiagnosis 5. The presence of co‐infection has also been reported in South‐east Asia, which may contribute further to under diagnosis of scrub typhus 6.

The clinical course is variable, ranging from spontaneous recovery without treatment to multiple organ failure with mortality ranging from 0 to 30% 7. The *Orientia* bacteria, inoculated via chigger saliva during feeding, infect dendritic cells and macrophages in the dermis underlying the characteristic eschar that can develop. This leads to local lymphogenous and subsequent hematogenous dissemination, involving endothelial cells and macrophages, which release soluble cell‐specific adhesion molecules and causes focal or disseminated vasculitis 5.

A review of scrub typhus in pregnancy reported on the total world literature: <100 cases with the pregnancy outcome reported in 18 years 2. Prospective cohort data suggests 8, 9 the adverse consequences are significant and include: maternal death 2.4% (2/82), miscarriage 17.3% (14/81), and poor neonatal outcomes (including stillbirth, preterm labor, small for gestational age, or low birth weight) 41.8% (28/67) 2. A recent systematic review reported a median mortality of 6.0% (range 0–70%) in 19,644 untreated patients, but specifically recommended the requirement for more data to clarify mortality in vulnerable mother–child populations 10.

## Case Report

**Figure 1:**
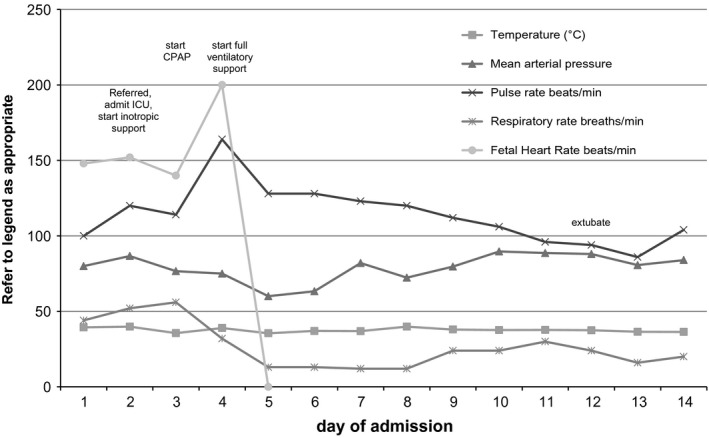
Vital signs during admission.

In December 2014, a 35‐year‐old woman, gravida 7, para 5, presented in the 31st gestational week to the clinic of Shoklo Malaria Research Unit (SMRU) in Maela refugee camp on the Thailand–Myanmar border. She complained of 6 days of fever, chills, rigors, headaches, nausea, cough, myalgia, joint pain, epigastric pain, and constipation for which she had self‐medicated with intravenous vitamin injections but denied taking antibiotics. Fever, tachycardia, and tachypnea were noted on admission (Fig. 1) and no other abnormalities were detected on a systems physical examination, specifically no hepatomegaly, eschar, rash, jaundice, or lymphadenopathy, and fetal growth was appropriate for gestation week. Her first antenatal visit in the 23rd gestational week had confirmed a viable pregnancy and no significant medical or obstetric problems.

At the refugee camp, the patient was treated for AUF with 1 g of intravenous ceftriaxone and fluid replacement, but mistakenly azithromycin was omitted. Before commencing antibiotics, a venipuncture sample of blood was taken for the analysis in the Mae Sot laboratory 60 km away and the results became available the same evening (Table 1). Confidence in the scrub typhus results were low as 1 year of field experience with this test in pregnancy at SMRU suggested the possibility of over diagnosis: 13.6% (3/22) RDT‐positive cases had a fourfold rise in IgM titer and none were PCR positive for scrub typhus 2. Initial clinical improvement was not maintained and at 24 h, persistent high‐grade fever, worsening dyspnea and the onset of bilateral crepitations and peripheral edema were thought to be due to pulmonary edema. Intravenous fluids were stopped, IV furosemide 40 mg was given, and antibiotic therapy was modified to ceftriaxone 2 g every 12 h, with oral azithromycin 500 mg once daily and oral metronidazole 500 mg every 8 h, being added. Due to the limited facilities at the field site, the patient was transferred to a higher level of care.

**Table 1:** Patient laboratory results [normal range, NR] as they emerged

	Abnormal results	Normal/Negative results
Admission Refugee Camp Day 1	1 –Urine stick: pH 6, glucose negative, ketones 2+, white blood cells (WBC) 1+, nitrite negative, protein 1+, hemoglobin 4+ 2 –Urine sediment microscopy: WBC 5.0/HPF, red blood cells 8.0/HPF, bacteria 1+, epithelial cells 0.5/HPF and uric acid crystals 1+, no WBC or RBC casts. 3 –Kidney ultrasound: dilation of the right renal pelvis, left kidney normal. 4 –Platelet count 125 × 109/L 5 –C‐reactive protein concentration >200 mg/L 6 –Creatinine kinase 199 [NR 26–140] units/L 7 –SD Bioline Tsutsugamushi rapid detection test: positive	1 –Malaria smear, hematocrit (32%) 2 –White blood cells count 5.1 × 109/L 3 –Differential count: neutrophils 4.3 × 109/L (84%); lymphocytes 0.4 × 109/L (8.0%); monocytes 0.4 × 109/L (8.0%) 4 –Hemoglobin 11.6 g/dL 5 –Hematocrit 32.9%
Admission Mae Sot Hospital (MSH) Day 2	1 –Arterial blood gas: pH 7.420, PaCO2 19.0 mmHg, base excess ‐12.3 2 –Potassium 3.3 [NR 3.3–5.1] mmol/L 3 –Sodium 133 [NR 130–148] mmol/L 4 –Platelets 74 [NR 140–450] × 109/L 5 –Scrub typhus IgM and IgG antibody: positive	1 –SaO2 99% on 3L O2 (nasal cannula) 2 –Serum aspartate transaminase 66 [NR 4–32] IU/L 3 –Alanine aminotransferase 61 [2–25] IU/L 4 –Alkaline phosphatase 184 [38–299] IU/L 5 –Protein total 5.4 [5.6–6.7] g/dL 6 –Albumin 2.7 g/dL [2.3–4.2] g/dL 7 –Creatinine 0.8 [NR 0.4–0.9] mg/dL 8 –Urea 8.54 [NR 3–11] mg/dL 9 –Blood dextrose: 78 mg/dL 10 –Urine culture: No significant growth 11 –Blood culture: No significant growth
MSH Day 3	1 –Dextrose 34 mg/dL	
MSH Day 4	1 –Platelets 14 [NR 140–450] × 109/L 2 –INR 1.25 [NR 0.9–1.2]	
5 weeks	1 –Scrub typhus PCR: positive	

In the Provincial Government Hospital in Mae Sot, Thailand, initial laboratory results were consistent with a severe mixed metabolic acidosis with respiratory alkalosis (Table 1). A provisional diagnosis of septic shock was given. Adjunctive therapy with dopamine, ceftriaxone at a higher dose (2 g every 12 h), and chloramphenicol (1 g every 8 h) were commenced to cover rickettsial infection. On day 3, continuous positive pressure ventilation was required, severe hypoglycemia (which remained persistent) and bleeding per vagina also occurred. In the early afternoon, the obstetrician prescribed hydrocortisone 100 mg 8 hourly for fetal lung maturation. On day 4, respiratory support was elevated to intubation and mechanical ventilation with a fraction of inspired oxygen FiO2 up to 100%, and further inotropic support with norepinephrine initiated. Unfortunately, further vaginal bleeding, decreased platelet count and coagulopathy ensued, and the fetal heart rate deteriorated with fetal death in utero occurring on day 4. Infusion with packed red blood cells and two units of fresh frozen plasma were given before labor induction with misoprostol and amniotomy. A stillborn baby was delivered vaginally on day 5. No autopsy of the neonate or placental pathology was done. After delivery, the patient improved and could be weaned from noradrenaline within 24 h. Repeated transfusions of packed red blood cells and platelets were required with the nidus of platelet count occurring on day 4. Ceftriaxone was continued for 10 days, chloramphenicol was switched after 5 days to doxycycline (100 mg every 12 h) and prescribed for 10 days. Due to bronchial spasticity, the patient was only extubated after 11 days of ventilatory support and therapy with dexamethasone and beta‐2‐mimetics. Three days after extubation, the patient was discharged back to SMRU.

Severe sacral ulceration (stage 4) required dressings and debridement over the next 2 weeks by which stage her eldest daughter had learnt how to do the dressings. She had made a full recovery. Five weeks after the patient had first presented the admission blood test for the PCR targeting gene that encodes the 47‐kDa outer‐membrane antigen highly specific for *O. tsutsugamushi* was reported as positive (the test is not routinely available).

## Discussion

Prolonged intensive care avoided a third trimester near miss maternal mortality but not fetal loss at 31+4 weeks gestation, after late presentation, misdiagnosis, and inappropriate first‐line antimicrobials. The hospital costs for this patient were equivalent to saving 10 lives by providing life‐saving cesarean section for five women. This typifies previous case reports of mortality from scrub typhus in pregnancy 5, 8.

Rapid clinical improvement after antibiotic administration is characteristic with doxycycline, the drug of choice for AUF in nonpregnant patients from settings endemic for scrub typhus and would favor a diagnosis of *O. tsutsugamushi* infection 6. For this case, rapid diagnostic tests (RDT) were employed for diagnosis and later confirmed by PCR. Although RDTs are simple to perform, they are expensive and not accurate enough to be relied upon 11. For definitive diagnosis, serology using paired sera is used. Although a PCR‐positive result is regarded as reliable, specimens must be obtained early in the course of the infection and such techniques are usually unavailable in resource‐limited settings in a clinically useful timeframe 12. Accurate, simple, and cost‐effective diagnostics for scrub typhus are therefore required. While they are ideal and may one day become available in rural clinics for scrub typhus, they remain experimental and expensive, so that even the tertiary referral hospital relied upon acute scrub typhus serology to diagnose this case 13.

Guidelines for nonmalaria AUF in pregnant women in low and middle income countries are lacking. Streamlining the treatment of AUF with short‐course doxycycline including in pregnancy is likely to be feasible in terms of cost and availability, although safety data is weak 5, 10, 14, 15. Availability of intravenous azithromycin is limited by cost, although good outcomes with intravenous treatments have been reported in pregnancy in two series 16, 17. Intravenous chloramphenicol, the antimicrobial most likely responsible for the acute recovery from infection, was correctly administered to save the mother's life not with‐standing concerns for gray baby syndrome 18. The cause of death in the neonate could not be established, but maternal multiorgan failure and/or *O. tsutsugamushi* vasculitis‐associated pathology of the placenta, possibly associated with thrombotic occlusions and/or coagulopathy cannot be excluded 19.

AUF in pregnant women is potentially life‐threatening and sepsis protocols are required for scrub typhus endemic settings. Treatment protocols for AUF in areas where scrub typhus is endemic should include: oral azithromycin (or doxycycline if azithromycin is unavailable 15) in uncomplicated AUF to prevent progress to severe disease; and preferably intravenous azithromycin (or chloramphenicol if azithromycin is unavailable) in severe disease. The aim of treatment in this case of scrub typhus was to save the mother and the fetus based on disease severity and gestation and the risks and benefits of the antibiotic. Evidence‐based guidelines for AUF in pregnant women in settings where the burden of vector‐borne disease is high are required. There is an urgent need for reliable field‐based RDTs for scrub typhus.

## Ethical Approval

Ethical approval to review SMRU hospital records was given by the Oxford Tropical Research Ethics Committee (OXTREC 28–09).

## Conflict of Interest

None declared.

## References

[1] Acestor, N. , R. Cooksey , P. N. Newton , D. Menard , P. J. Guerin , J. Nakagawa , et al. 2012 Mapping the aetiology of non‐malarial febrile illness in Southeast Asia through a systematic review–terra incognita impairing treatment policies. PLoS One 7:e44269. 22970193 10.1371/journal.pone.0044269 PMC3435412

[2] McGready, R. , J. A. Prakash , S. J. Benjamin , W. Watthanaworawit , T. Anantatat , A. Tanganuchitcharnchai , et al. 2014 Pregnancy outcome in relation to treatment of murine typhus and scrub typhus infection: a fever cohort and a case series analysis. PLoS Negl. Trop. Dis. 8:e3327. 25412503 10.1371/journal.pntd.0003327 PMC4238995

[3] Koh, G. C. , R. J. Maude , D. H. Paris , P. N. Newton , and S. D. Blacksell . 2010 Diagnosis of scrub typhus. Am. J. Trop. Med. Hyg. 82:368–370. 20207857 10.4269/ajtmh.2010.09-0233 PMC2829893

[4] Ellis, R. D. , M. M. Fukuda , P. McDaniel , K. Welch , A. Nisalak , C. K. Murray , et al. 2006 Causes of fever in adults on the Thai‐Myanmar border. Am. J. Trop. Med. Hyg. 74:108–113. 16407353

[5] Paris, D. H. , T. R. Shelite , N. P. Day , and D. H. Walker . 2013 Unresolved problems related to scrub typhus: a seriously neglected life‐threatening disease. Am. J. Trop. Med. Hyg. 89:301–307. 23926142 10.4269/ajtmh.13-0064 PMC3741252

[6] Mayxay, M. , J. Castonguay‐Vanier , V. Chansamouth , A. Dubot‐Pérès , D. H. Paris , R. Phetsouvanh , et al. 2013 Causes of non‐malarial fever in Laos: a prospective study. Lancet Glob. Health 1:e46–e54. 24748368 10.1016/S2214-109X(13)70008-1 PMC3986032

[7] Rajapakse, S. , C. Rodrigo , and D. Fernando . 2012 Scrub typhus: pathophysiology, clinical manifestations and prognosis. Asian Pac. J. Trop. Med. 5:261–264. 22449515 10.1016/S1995-7645(12)60036-4

[8] Sengupta, M. , S. Benjamin , and J. A. Prakash . 2014 Scrub typhus continues to be a threat in pregnancy. J. Gynaecol. Obstet. 127:212. 10.1016/j.ijgo.2014.06.014 25109770

[9] McGready, R. , E. A. Ashley , V. Wuthiekanun , S. O. Tan , M. Pimanpanarak , S. J. Viladpai‐Nguen , et al. 2010 Arthropod borne disease: the leading cause of fever in pregnancy on the Thai‐Burmese border. PLoS Negl. Trop. Dis. 4:e888. 21103369 10.1371/journal.pntd.0000888 PMC2982829

[10] Taylor, A. J. , D. H. Paris , and P. N. Newton . 2015 A systematic review of mortality from untreated scrub typhus (Orientia tsutsugamushi). PLoS Negl. Trop. Dis. 9:e0003971. 26274584 10.1371/journal.pntd.0003971 PMC4537241

[11] Watthanaworawit, W. , P. Turner , C. Turner , A. Tanganuchitcharnchai , S. Jintaworn , B. Hanboonkunupakarn , et al. 2015 Diagnostic accuracy assessment of immunochromatographic tests for the rapid detection of antibodies against orientia tsutsugamushi using paired acute and convalescent specimens. Am. J. Trop. Med. Hyg. 93:1168–1171. 26458778 10.4269/ajtmh.15-0435 PMC4674230

[12] Watthanaworawit, W. , P. Turner , C. Turner , A. Tanganuchitcharnchai , A. L. Richards , K. M. Bourzac , et al. 2013 A prospective evaluation of real‐time PCR assays for the detection of Orientia tsutsugamushi and Rickettsia spp. for early diagnosis of rickettsial infections during the acute phase of undifferentiated febrile illness. Am. J. Trop. Med. Hyg. 89:308–310. 23732256 10.4269/ajtmh.12-0600 PMC3741253

[13] Kingston, H. W. , S. D. Blacksell , A. Tanganuchitcharnchai , A. Laongnualpanich , B. Basnyat , N. P. Day , et al. 2015 Comparative accuracy of the InBios scrub typhus detect IgM rapid test for the detection of IgM antibodies by using conventional serology. Clin. Vaccine Immunol. 22:1130–1132. 26291089 10.1128/CVI.00390-15 PMC4580738

[14] Fang, Y. , Z. Huang , C. Tu , L. Zhang , D. Ye , and B. P. Zhu . 2012 Meta‐analysis of drug treatment for scrub typhus in Asia. Intern. Med. 51:2313–2320 Epub 2012/09/15. 22975540 10.2169/internalmedicine.51.7816

[15] Cross, R. , C. Ling , N. P. Day , R. McGready , and D. H. Paris . 2016 Revisiting doxycycline in pregnancy and early childhood ‐ time to rebuild its reputation? Expert Opin. Drug Saf. 15:367–382. 26680308 10.1517/14740338.2016.1133584 PMC4898140

[16] Raman, S. , V. P. Krishna , Manjunath , H. Singh , S. Shrivastava , V. Singh , et al. 2014 Analysis of two outbreaks of scrub typhus in rajasthan: a clinico‐epidemiological study. J. Assoc. Physicians India 62:24–29. 26259419

[17] Poomalar, G. K. , R. Rekha ., 2014 A case series of scrub typhus in obstetrics. J. Clin. Diagn. Res. 8:OR01–OR03. 25653996 10.7860/JCDR/2014/9718.5258 PMC4316302

[18] Amstey, M. S. 2000 Chloramphenicol therapy in pregnancy. Clin. Infect. Dis. 30:237. 10619781 10.1086/313582

[19] Paris, D. H. , R. Phetsouvanh , A. Tanganuchitcharnchai , M. Jones , K. Jenjaroen , M. Vongsouvath , et al. 2012 Orientia tsutsugamushi in human scrub typhus eschars shows tropism for dendritic cells and monocytes rather than endothelium. PLoS Negl. Trop. Dis. 6:e1466. 22253938 10.1371/journal.pntd.0001466 PMC3254662

